# Modeling of Distributed Sensing of Elastic Waves by Fiber-Optic Interferometry

**DOI:** 10.3390/s16091433

**Published:** 2016-09-06

**Authors:** Just Agbodjan Prince, Franz Kohl, Thilo Sauter

**Affiliations:** 1 Center for Integrated Sensor Systems, Danube University Krems, Viktor Kaplan Straße 2, 2700 Wr. Neustadt, Austria; agbodjan@iiss.at (J.A.P.); franz.kohl@tuwien.ac.at (F.K.); 2 Institute of Computer Technology, Vienna University of Technology, Gußhausstraße 27-29/384, 1040 Vienna, Austria

**Keywords:** strain sensor, interferometry, fiber optic

## Abstract

This paper deals with the transduction of strain accompanying elastic waves in solids by firmly attached optical fibers. Stretching sections of optical fibers changes the time required by guided light to pass such sections. Exploiting interferometric techniques, highly sensitive fiber-optic strain transducers are feasible based on this fiber-intrinsic effect. The impact on the actual strain conversion of the fiber segment’s shape and size, as well as its inclination to the elastic wavefront is studied. FEM analyses show that severe distortions of the interferometric response occur when the attached fiber length spans a noticeable fraction of the elastic wavelength. Analytical models of strain transduction are presented for typical transducer shapes. They are used to compute input-output relationships for the transduction of narrow-band strain pulses as a function of the mechanical wavelength. The described approach applies to many transducers depending on the distributed interaction with the investigated object.

## 1. Introduction

Ultrasonic waves are convenient means to investigate the structural integrity of extended objects [1,2,3,4,5,6,7,8]. Elastic waves in solids may propagate over considerable distances as various modes of bulk waves, Rayleigh waves [9], Love waves [10] and Lamb waves [11], for example. Lamb waves are easily excited in the device under test by attached piezoelectric actuators [3,4,12]. Damages in the investigated object influence ultrasonic waves’ propagation or excite secondary partial waves. Elastic waves may be visualized in a contactless manner by laser Doppler vibrometry, but they are typically probed with attached transducers that are sensitive to strain variations induced by elastic waves in the specimen [13]. A technically elegant way to perform such investigations is to use a single piezoelectric transducer for both the excitation and detection of elastic waves [12]. However, piezoelectric strain transducers suffer from interferences by electromagnetic fields generated, for example, during piezoelectric actuation of the elastic waves to be detected [14,15,16]. Fiber-optic strain transducers, on the other hand, are beneficial because of their nearly perfect immunity to external electromagnetic fields [17,18]. Even more, in contrast to piezoelectric strain transducers, the attached lightweight optical waveguide has no relevant mechanical resonances and causes negligible mechanical loading to the investigated specimen, which simplifies the analysis of the system behavior.

Fiber Bragg grating (FBG) transducers are very common devices for the transduction of elastic waves [8]. FBGs make use of localized modulations of the characteristics of an optical waveguide. Periodic modulation of waveguide parameters in the axial direction form a grating that shapes the spectral transmission characteristic of such modified fiber sections. Subsequently, strain applied along the FBG region results in a shift of this spectral transmission characteristic, which can be evaluated with a suitable optoelectronic signal processing system.

As an alternative approach, interferometric fiber-optic transducers rely on the strain dependence of fiber-intrinsic properties [19,20]. Such transducers comprise typically two fiber arms, i.e., a segment of an optical waveguide that is firmly attached to the investigated specimen and an unperturbed reference fiber. They are complemented by an optical arrangement that ensures interference between the light waves of measurement and reference waveguide. Pronounced directional, as well as perfect omnidirectional detection characteristics can be achieved, depending on the shape of the interacting fiber segment. At high ultrasound frequencies, however, the dimensions of all kinds of attached transducers may become comparable to the wavelength of the elastic waves, which may impair the fidelity of the readout signal. The bulk of the paper is devoted to a study of this generic situation, and intrinsic fiber optic strain transduction is well suited for such a study.

Some fundamentals of strain conversion with optical waveguides are sketched in Section 2. Section 3 describes a finite element model aiming at the evaluation of transduction distortions. Furthermore, an analytical model is presented enabling conversion efficiency studies for various configurations of distributed fiber-optic strain transduction. The related results will be presented in Section 4.

## 2. Interferometric Strain Transduction

### 2.1. All Fiber Interferometer

**Figure 1:**
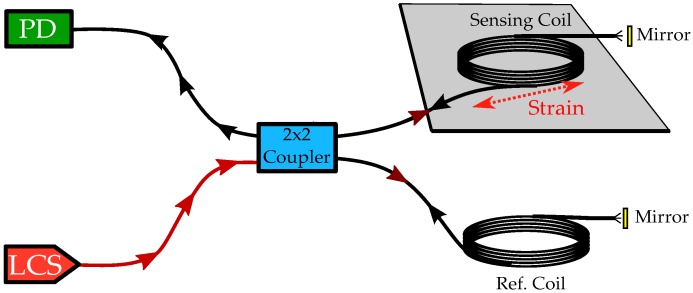
Fiber-optic Michelson interferometer for strain transduction. The sensing fiber coil is bonded to the specimen, which is strained by an elastic wave. The produced light from LCS and the light reflected at the fiber’s end are split/recombined in the coupler.

The fiber-optic transducer illustrated in Figure 1 relies on a photodetector (PD), a sufficiently coherent light source (LCS) and a 2 × 2 fiber coupler together forming a Michelson interferometer. The coupler links the sensing and the reference fiber segment, both at the secondary ports to the light source branch and the detector branch on the primary side. Nearly monochromatic light generated by the source is split in the coupler and guided, e.g., towards two single-mode fiber coils. A segment of the sensing fiber is tightly attached to the specimen that exhibits elastic deformation. Therefore, changes of the optical path length of this wave guide will occur in relation to the reference segment. Through the 2 × 2 coupler, part of the reflected waves of both the reference and sensing branch is guided to the PD. Interference of the partial waves takes place at the photodetector and can be evaluated by standard techniques.

### 2.2. Fiber-Intrinsic Strain Effects

The elastic wave propagating in the plate causes oscillatory material particle movement, which influences the attached optical waveguide fiber. Deformation of the fiber changes its length *L*, diameter *D* and, because of the photo-elastic effect, the effective refraction index $n_{\mathrm{eff}}\left(\lambda \right)$ of the fiber material. The variation of the fiber cross-section size has been shown to have negligible impact on light guidance compared to concurrent length changes [21].

The optical path length of a fiber segment is the product of the effective refractive index of the fiber $n_{\mathrm{eff}}$ and its physical length *L*. Changes in the refractive index $▵n_{\mathrm{eff}}$, as well as changes in the optical fiber length ▵L modulate the optical path length. Due to reflection at the end faces of the measurement and reference waveguide, the optical phase shift φ between light emission and re-entrance at the secondary ports of the coupler is given by:

(1) $$\varphi =4\pi n_{\mathrm{eff}}L/\lambda_{0},$$

when the interferometer is in balance. $\lambda_{0}$ denotes the free space wavelength of the guided light. Uniform axial strain caused by an elastic wave along a predetermined interaction path, which is defined by the attached fiber segment itself, changes $n_{\mathrm{eff}}$, as well as *L*. Hence, the total propagation delay is converted into changes of phase Δφ,

(2) $$\Delta \varphi \propto \Delta \left(n_{\mathrm{eff}}\;L\right)=n_{\mathrm{eff}}\;\Delta L\;+L\;\Delta n_{\mathrm{eff}}.$$

In general, the variation of the fiber length is given by the path integral of the local fiber axis strain.

Using the average strain $\bar{\varepsilon }=\int_{L}\varepsilon \;dl$ of the fiber, the first term on the right hand side of Equation (2) reads:

(3) $$n_{\mathrm{eff}}\;▵L=n_{\mathrm{eff}}\;\bar{\varepsilon }\;L$$

while $▵n_{\mathrm{eff}}$ can be computed from [17]:

(4) $$▵n_{eff}=0.5\;n_{0}^{3}\;\bar{\varepsilon }\;\left(\nu \;\left(P_{11}+P_{12}\right)-P_{12}\right)\;\approx -0.314\;\bar{\varepsilon }.$$

Here, $P_{ij}$ and *ν* are the photo-elastic constants and the Poisson number of silica, respectively. According to [17], the relevant photo-elastic tensor elements are $P_{11}=+0.121$ and $P_{12}=+0.270$; $n_{0}=1.456$ is the refraction index of the relaxed fiber at the preferred wavelength of 1310 nm, and ν=0.17. Hence, Equation (2) becomes:

(5) $$▵\left(n_{\mathrm{eff}}\;L\right)\;=\;0.686\;n_{\mathrm{eff}}\;\bar{\varepsilon }\;L.$$

Roughly speaking, the index change compensates nearly 30% of the propagation delay caused by the geometric stretch of the fiber length alone [21]. However, the simple relation Equation (5) conceals a rich variety of sensing peculiarities. As the sensitivity of the interferometric fiber-optic transducer increases with the interacting length of the fiber, it is advisable to shape the attached fiber segment into a flat, multi-turn spiral form. Then, the local angle *θ* between the fiber axis and strain orientation at the plate surface varies, and the sensitivity to local strain is governed by the cosine of *θ*. Furthermore, the extension of the spiral coil may easily exceed the wavelength $\lambda_{M}$ of the elastic waves, leaving us with the problem of distributed detection and related signal distortions by convolution of the detector characteristic with the strain field of the exciting wave. For this study, it has to be assured whether or not the repercussion of the attached silica fiber on the elastic perturbation in the specimen is negligible at least to a first order approximation.

The main questions to be answered by finite element analysis (FEA) are: (i) What is the conversion efficiency of the transducer? (ii) Are there severe repercussion effects of the attached silica fiber coil to the investigated plate? (iii) What detection characteristics result, considering typical wavelengths of ultrasound diagnostics and the design of the fiber segment (spiral coil or straight)?

### 2.3. Previous Work

In prior work, we have established a small-scale FEA suite for studies of fiber-optic Lamb wave transduction [22]. Fundamental symmetric Lamb waves were excited by suitable boundary conditions, and the propagation of related wave pulses could be observed with transient FEA. Such small-scale models are beneficial with respect to computational efforts, but multiple reflections at the model boundaries reduce the useful time span of such studies. However, using this approach, it was possible to demonstrate that mechanical reactions owing to an attached fiber-optic transducer are only weak [22]. These efforts focused specifically on attached fiber segments shaped in the form of a flat spiral, which enables omnidirectional transduction.

In order to verify the fiber optical transducer function, a FEM model comprising a thin aluminum plate with an attached five-turn spiral monomode optical fiber was assembled. Aluminum 3003-H18 material (ρ=2730$\;\mathrm{kg}/m^{3}$, E=69GPa, ν= 0.33, loss factor 0.001) was used for the plate of thickness d=0.75 mm and a 4.5 cm × 12.5 cm area. A silica monomode fiber with 125µm diameter, protected by an acrylic coat of 250µm outer diameter was slightly adapted to obtain an economic simulation model. For fast simulations, the circular fiber cross-section was approximated by a square shape with a side length of 250µm, and the material parameters E=14.3 GPa, ν=0.22 and $\rho =431.95\;\mathrm{kg}/m^{3}$ were adjusted according to the cross-section ratios of the fiber, coating and model. A single-sine burst pressure signal:

(6) $$p\left(t\right)=\sin\left(2\pi f_{0}t\right)\cdot {\left[\sin\left(\pi f_{0}t/N)\right)\right]}^{2},\;0<t<N/f_{0},$$

with N=2 was imposed parallel to the *x*-axis at one edge face of the plate that is oriented perpendicular to this axis, where $f_{0}=100\;\mathrm{kHz}$ denotes the center frequency of the wave packet. As the resulting dilatational perturbation excites exclusively the compressional $S_{0}$ Lamb wave mode, the related strain is also oriented parallel to the *x*-axis and related to the *x*-derivative of the *x*-displacement. The simulated *y*-displacement induced by the attached fiber coil turned out to be in the order of 1% of the primary *x*-displacement.

**Figure 2:**
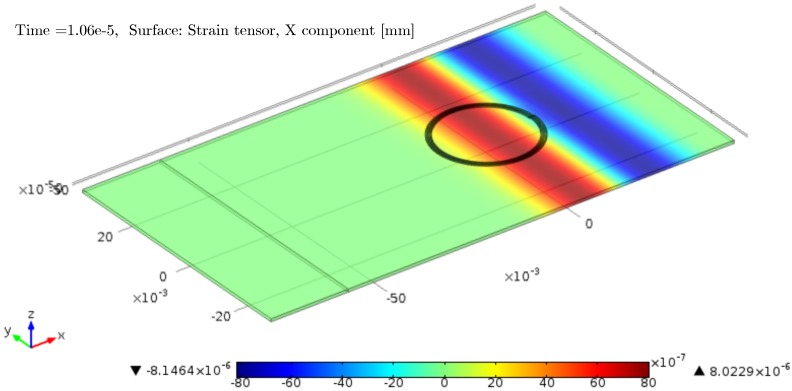
FEM result for comprising a five-turn spiral-wound fiber replacement of outer diameter 2R=25mm on an aluminum plate. Color code: *x*-component of $S_{0}$ wave strain at 10 µs.

Figure 2 displays a representative snapshot of the simulated surface strain taken at 10.6 µs of elapsed time.

**Figure 3:**
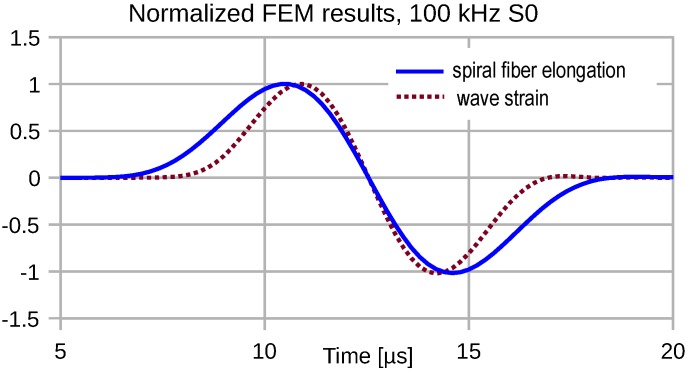
Simulated elongation of a five-turn fiber spiral in response to the shown single-sine strain pulse excitation with $f_{0}=100\;\mathrm{kHz}$.

The interferometric signal was derived from the total length change of the edge length of the spiraled fiber. This strain conversion corresponds to an integration along the fiber axis of the local fiber stretching *ε* by the local strain given by $\varepsilon^{2}=\vec{a}\cdot \underline{G}\cdot \vec{a}$, where $\underline{G}$ denotes Green’s deformation tensor and $\vec{a}$ the unit vector in the axial direction. Interferometric conversion by an attached fiber involves a spatial convolution operation of the propagating strain field with the spatial fiber sensitivity characteristic. Of course, the convolution result is directly mapped to the time domain in the case of negligible velocity dispersion. Fortunately, FEA results for compressional waves revealed a nearly constant propagation velocity for the simulated frequency band centered at 100 kHz. The actual propagation velocities could be estimated from the further progress of the strain pattern shown in Figure 2. At $f_{0}=100\;\mathrm{kHz}$, a group velocity of 5250 m/s results. The corresponding transducer response is shown in Figure 3 together with the wave strain variation at the center of the spiral to emphasize the significant temporal stretch of the transducer output.

Based on the estimated propagation velocity and the actual size of the fiber coil, the strain response can also be computed by an analytical model, which is described in detail below. Instead of a spiral coil, the analytical model assumes a semicircular shape of the fiber, and the results are then multiplied by a factor of 10 for comparison with the FEM results for a five-turn fiber spiral.

### 2.4. Remaining Scientific Topics

The finite extension of the region of contact between the strain transducer and the carrier of the elastic perturbation may cause severe distortions of transduced strain pulses, as Figure 3 demonstrates. The transduction of strain pulses depends on the actual transducer shape governing the orientation of the attached fiber segments with respect to the wavefront of the guided elastic wave. In the following, the pulse transduction distortions are studied for some important configurations of the fiber-optic strain transducer using the approved FEA approach.

The transducer output pulses may show severe distortions for short wavelengths $\lambda_{M}$, which requires an appropriate measure for the conversion efficiency. As the elastic wave-based diagnostic usually depends on wave pulses, the detectability of such pulses is a key issue. The ratio of the distorted transducer output pulse energy over the strain pulse energy proved to be a useful quantity for this purpose. On the basis of this performance figure, a sort of transfer function can be computed, based on a fixed strain pulse shape, but varying center frequency $f_{0}$. The presented approach uses the popular sine-burst wave pulse family and the assumption of a constant propagation velocity.

## 3. Modeling of Specific Optical Fiber Configurations

**Figure 4:**
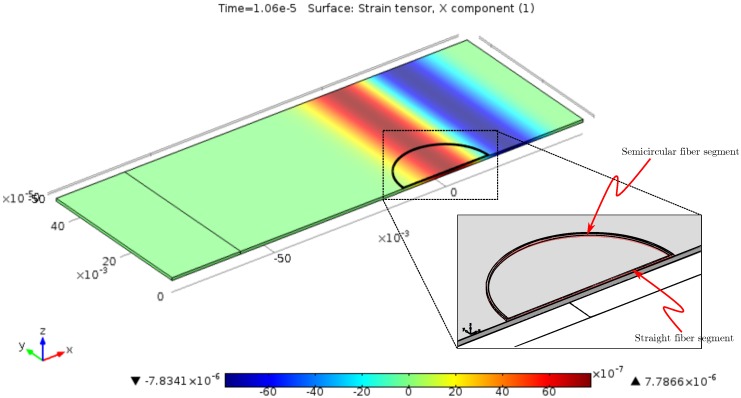
FEM model exhibiting a snapshot of a 100-kHz plane $S_{0}$ strain pulse, with the aluminum plate as the monitoring structure. The attached sensor fiber segment is at the same time a straight piece and a semicircle.

A modified FEM model according to Figure 4 was designed to simulate the response of circular and straight fiber optic transducer segments simultaneously. Instead of a spiral wound fiber, the aluminum plate of 0.75mm thickness bears a half-circle-shaped fiber imitation adjacent to the symmetry plane of the model that is complemented by a straight segment, which spans the diameter of the circle. Assuming negligible loading effects, the model enables the computation of the elongations of circular, as well as straight transducer configurations.

### Analytical Model for Input-Output Analyses

Guided waves are typically produced by piezoelectric ceramic devices glued to the plate. Depending on the device shape and the distance between the source and sensing transducer, various shapes of the guided wavefront are observed. In this section, some fundamental consequences of the guided wave detection with circular fiber-optic transducers are discussed. To quickly cover the typical convolution characteristics for plane and circular crested plate waves, as well, an analytical model of fiber optic strain conversion has been established. This model is capable of examining wave strain conversion by a straight piece of fiber inclined at a preselected angle to the propagation direction. Furthermore, transduction by a circular bent fiber of radius *R* can be treated for plane and circular crested wavefronts, where a center to center distance d=2R was chosen for explicit results in the latter case. At last, the analytical approach is also used to model the frequency dependence of the strain pulse conversion.

To enable a straightforward treatment of circular and straight transducer fiber, plane Lamb waves are given preference. Fortunately, at large distances from any actuator, initially curved wavefronts become nearly flat. Consequently, the obtained behavior is also typical for circular fiber transducers located far from the site of arbitrarily-shaped actuators.

**Figure 5:**
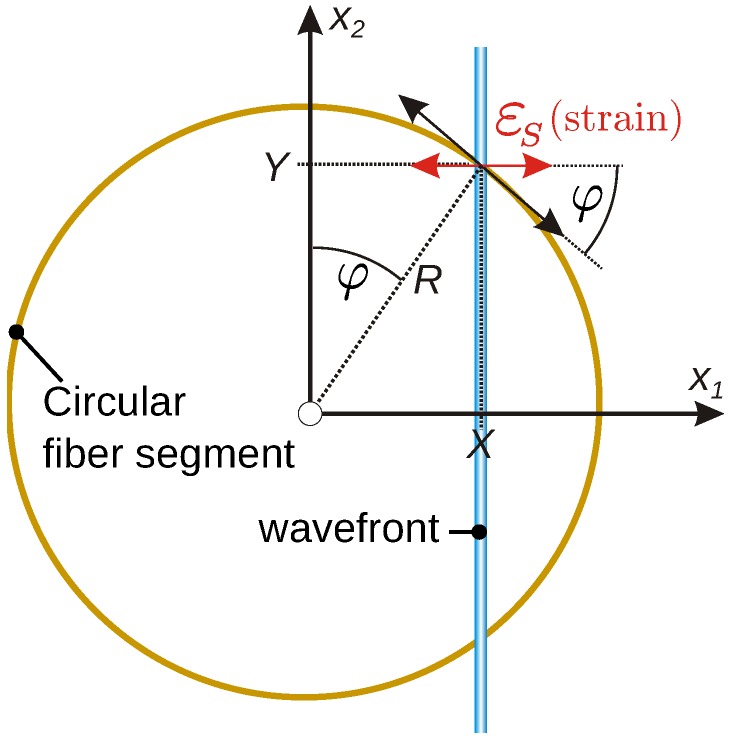
Derivation of the strain conversion by circular fibers for plane compressional waves.

Figure 5 explains the interaction of a plane wavefront (colored vertical bar) with a circularly bent fiber. The pure compressional wave is assumed to propagate along the $x_{1}$-direction, and therefore, the wave related strain $\varepsilon_{S}$ at the plate surface is also parallel to $x_{1}$. The wavefront under consideration crosses the circle at a distance X<R from the center. Then, the cosine of the crossing angle φ between surface strain orientation and the tangent to the fiber circle is given by:

$$\cos\varphi =Y/R=\sqrt{1-X^{2}/R^{2}}.$$

Neglecting any repercussion on the wave by the attached fiber, the axial strain of the fiber at the wavefront cross-over location is given by $\varepsilon_{S}\cdot \cos\left[\varphi \left(X\right)\right]$. Simply put, $\cos[\varphi \left(x_{1}\right)]$ is the spatial sensitivity characteristic of the circular fiber in the case of plane compressional waves. To obtain the total length change of the circular fiber at a specified moment $t_{0}$, the effect of the momentary snapshot of the wave strain $\varepsilon_{S}\left(x_{1},\;t_{0}\right)$ has to be integrated along the full circular path:

(7) $$2\cdot \int_{-R}^{R}\varepsilon_{S}\left(x_{1},t_{0}\right)\cdot \cos\left[\varphi \left(x_{1}\right)\right]dx_{1},$$

where we took advantage of the $x_{1}$-axis symmetry. To compute the time dependent response to a compressional wave, the propagation of the wavefront must be considered. This is most easily done for stable wave packet shape functions, i.e., non-dispersive waves. Then, a time shift dτ corresponds to a wavefront propagation distance $dx_{1}=v_{\mathrm{PH}}\cdot d\tau .$ In other words, the strain pattern is shifted by $dx_{1}$ with respect to the circular integration path or the sensitivity function is shifted against the snapshot of the wave strain pulse by the same amount. The complete response of the circular fiber to a compact wave packet can be computed from a convolution integral of the momentary spatial shape of the strain pulse and the fiber sensitivity curve in the space domain. If the strain pulse has a duration of $\tau_{P}$, its spatial extension measures $l=\tau_{P}\cdot v_{PH}$, and the total convolution response requires a shift distance ≥(l+2R). Hence,

(8) $$f\left(x\right)\int_{x_{0}-R}^{x_{0}+l+R}\;\varepsilon_{S}\left(\xi \right)w\left(x-\xi \right)d\xi ,$$

where $\varepsilon_{S}\left(\xi \right)$ denotes the strain pulse snapshot in the space domain, starting at $\xi =x_{0}$, and:

(9) $$w\left(x\right)=2\sqrt{1-x^{2}/R^{2}},\;x^{2}<R^{2}$$

is the sensitivity function of the circular bent fiber, where the factor of two results from the two intersections of the wavefront and the full circle.

For circular crested waves emerging from a center at distance *d* from the center of the fiber circle, the window functions for radial and tangential (hoop) strain conversion, $w_{r}\left(x\right)$ and $w_{t}\left(x\right)$, follow from basic geometrical relations as:

(10) $$w_{r}\left(x\right)=2d\cdot \sqrt{1-x^{2}/R^{2}}/\sqrt{d^{2}+R^{2}-2dx},\;x^{2}<R^{2}$$

and:

(11) $$w_{t}\left(x\right)=\left(2xd-R^{2}\right)/\sqrt{d^{2}+R^{2}-2dx},\;x^{2}<R^{2},$$

respectively. While the radial strain accompanying circular crested elastic waves is given by the gradient of wave displacement, the hoop strain is in proportion to the radial wave displacement divided by the radius of the wavefront curvature. Extrema of both $w_{t}\left(x\right)$ and $w_{r}\left(x\right)$ occur where the wavefront is perpendicular or parallel to the fiber circle.

**Figure 6:**
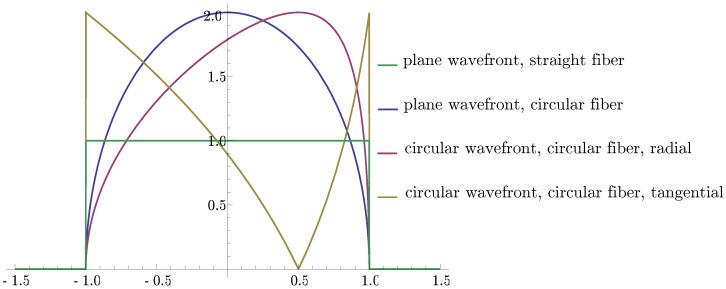
Normalized window functions for strain conversion by straight $\left(\Theta =0\right)$ and circular optical fibers. Plane and circular wavefronts are considered. The circular crested waves emerge at a distance d=2R.

For a single straight fiber of length 2R oriented in the propagation direction of a plane compressional wave, one obtains:

(12) $$w_{s}\left(x\right)=1,\;x^{2}<R^{2},$$

whereas for the same fiber segment, but inclined at an angle Θ relative to the propagation direction:

(13) $$w_{i}\left(x\right)=\cos\Theta ,\;x^{2}<{\left(R\cdot \cos\Theta \right)}^{2}$$

follows. Figure 6 depicts window functions over x/R for some of these cases, showing the strong influence of both the active transducer segment and the wavefront shapes on the strain conversion.

It is obvious that the convolution integral of Equation (8) extends the response $f\left(x\right)$ by 2R or 2RcosΘ compared to the strain pulse $\varepsilon_{s}\left(x\right)$ for any window function of Figure 6.

For dispersion-free waves, the argument of the response has to be replaced to get the temporal response, i.e., $g\left(t\right)=f\left(x/v_{\mathrm{PH}}\right)$, that is then prolonged by $2R/v_{\mathrm{PH}}$ compared to the duration of the strain pulse at any fixed position. For strain pulses accompanying dispersive modes of elastic waves, Equation (8) has to be modified by replacing $\varepsilon_{S}\left(\xi \right)$ with $\varepsilon_{S}\left(\xi ,t\right)$ to account for the temporal development of the wave strain.

## 4. Results

### 4.1. Transduction Distortions

**Figure 7:**
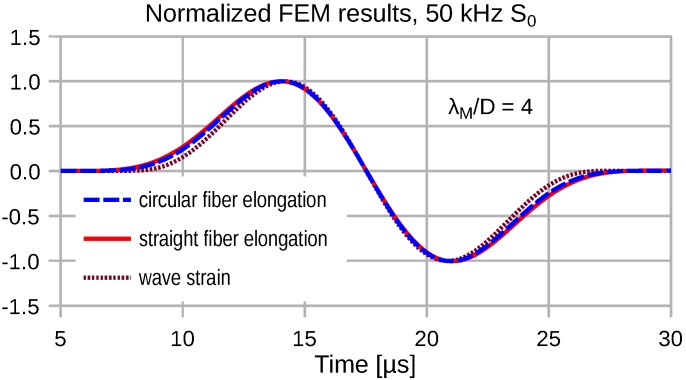
Low frequency transduction of plane wave strain by a circular and a straight fiber optic transducer. The fiber extent *D* is $\lambda_{M}/4$ with $\lambda_{M}=v_{\mathrm{PH}}/f_{0}$.

**Figure 8:**
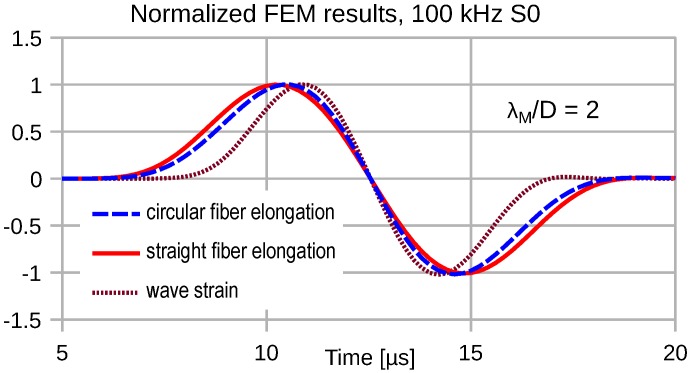
Plane wave $S_{0}$(100 kHz) strain and converted signals for both transducer elongation configurations for $\lambda_{M}=2D$.

**Figure 9:**
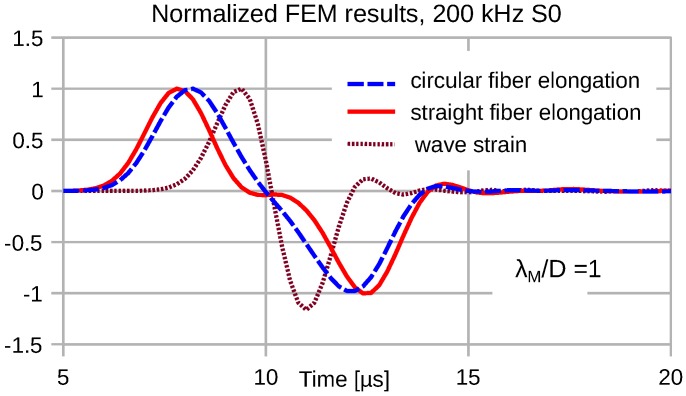
Time dependence of plane wave strain and circular/straight transducer output signals for $\lambda_{M}=D$.

**Figure 10:**
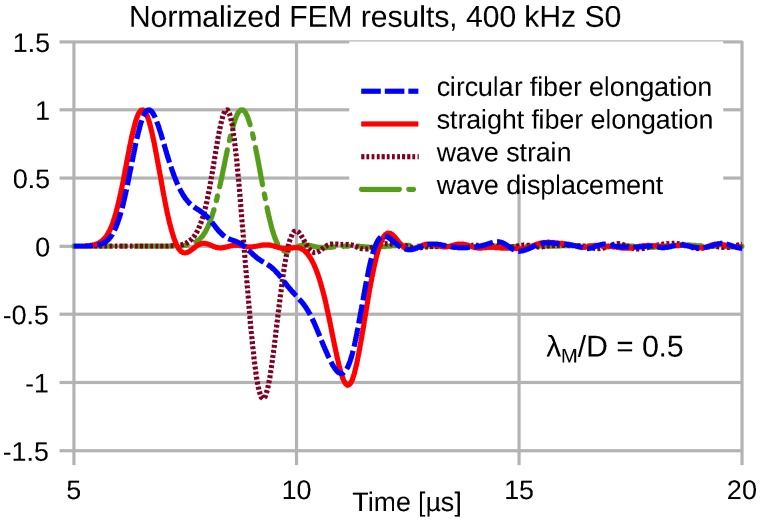
Extremely distorted outputs of straight and circular fiber transducers in response to the indicated plane wave strain and displacement result for short mechanical wavelengths $2\lambda_{M}=D$.

Due to the distributed transduction of the wave strain, we observe a strong frequency dependence of the transduction signal fidelity. At low frequencies, where $\lambda_{M}\gg 2R=D$, the transducer output signal exhibits a minor temporal stretch compared to the wave strain. Figure 7, Figure 8, Figure 9 and Figure 10 depict the input wave strain for an $S_{0}$ sine-burst wave of pulse length N=2 (see Equation (6)) and the related transducer outputs for both a straight and a semicircular shape of the attached fiber. Both fiber segments extend over a distance *D* along the propagation direction of the wave. In order to display the signal distortions due to the distributed sensing clearly, all displayed FEM results are normalized with respect to their magnitudes.

For $\lambda_{M}=2D$ the temporal stretch of the transducer signals becomes obvious in Figure 8, where the straight fiber causes the larger temporal stretch.

For $\lambda_{M}=D$, the transducer signal shapes exhibit severe deviations from the wave strain time dependence; see Figure 9. The simulated wave strain exhibits a trailing undulation as a result of the non-vanishing dispersion of the fundamental compressional mode.

Figure 10 shows FEM results for $f_{0}=$ 400 kHz, which corresponds to $2\lambda_{M}=D$. The integral over the wave strain signal that equals wave displacement is shown to illustrate its affinity with the convolution of wave strain with the two-step functions included in Equation (12), i.e., the processing by the straight transducer. The period between the positive and negative peak of the elongation signal corresponds to the passage time of the pulse $D/v_{\mathrm{ph}}$, i.e., $25\;\mathrm{mm}/5.25\;\;\mathrm{km}s^{-1}$= 4.76 µs.

### 4.2. Pulse Energy Transduction

#### 4.2.1. Wavelength Dependence of Straight Fiber Transducers

It seems instructive to consider conversion efficiencies for the fixed displacement magnitude, as well as the fixed strain magnitude of the applied waves. Constant strain means that the displacement magnitude increases proportionally to the wavelength $\lambda_{M}$ of the elastic wave, whereas for constant displacement amplitude, the strain amplitude varies in proportion to $\lambda_{M}^{-1}$.

To derive a figure of merit for the transduction in the case of severe signal distortions, there is no generally applicable procedure available. If signal fidelity is not feasible, a criterion for the detection threshold could be of primary interest. For this purpose, the peak-signal ratio of the transducer signal and the exciting strain signal may be used, for example. Such performance figures, however, are vulnerable in the case of significant measurement noise. We, therefore, suggest to relate the mean pulse energy of the distorted transducer signal to the mean pulse energy of the exciting signal as an appropriate measure of the transducer efficiency.

**Figure 11:**
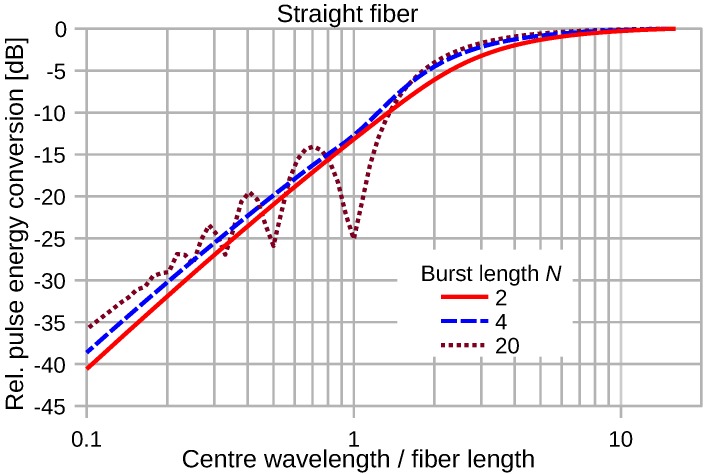
Normalized energy conversion of various sine-burst pulses by a straight fiber segment as a function of $\lambda_{M}/D$. The wave strain magnitude is kept constant.

Figure 11 depicts analytically-computed energy conversion efficiencies of an attached straight optical waveguide of length *D* for various plane wave pulses based on Equations (8) and (12). Three types of sine-burst pulses according to Equation (6) with *N* = 2, 4 and 20 were considered. At long wavelengths of the elastic wave, the wave strain does not vary significantly along the attached fiber, and the energy of the output signal pulse saturates. Towards short wavelengths, the wave strain varies along the attached fiber segment, and severe convolution distortions take place, leading to a decrease of the conversion efficiency by about 30 dB/decade. For sufficiently long sine-burst strains, e.g., for N=20, the conversion efficiency exhibits marked notches when the length of the attached fiber measures a multiple of the center wavelength of the elastic wave, i.e., D/λ approaches an integer value.

**Figure 12:**
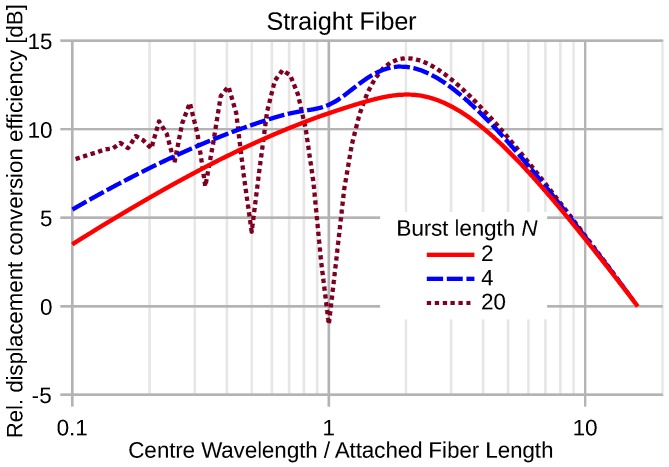
Normalized energy conversion of various sine-burst pulses by a straight fiber segment as a function of $\lambda_{M}/D$. The wave displacement magnitude is kept constant.

Figure 12 depicts the conversion efficiency for elastic waves of a straight segment of attached fiber when the maximum displacement of the wave pulse is kept constant. Towards short wavelengths $\lambda_{M}\ll D$, the conversion efficiency decreases with decreasing wavelength of the elastic wave by about 10 dB/decade due to severe convolution distortions, which are not completely compensated by the increasing peak strain. Towards very long wavelengths, the squared signal magnitude decreases with increasing wavelength by 20 dB/decade according to the decrease of the wave strain.

**Figure 13:**
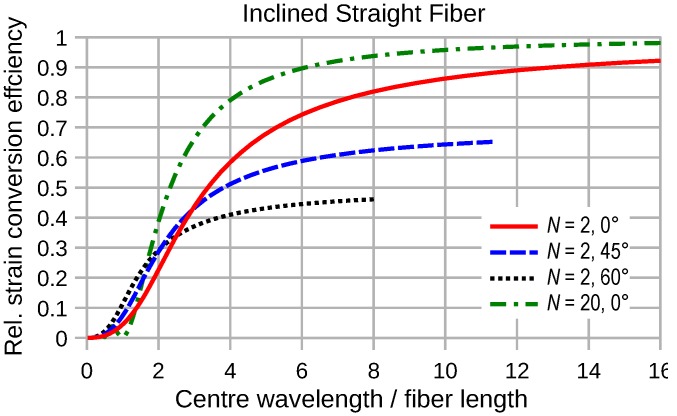
Conversion efficiency of a straight fiber segment oriented parallel or inclined to the wave propagation direction as a function of $\lambda_{M}/D$.

The effect of the inclined incidence of elastic waves on the straight transducer efficiency can be seen from Figure 13. Any tilt between the fiber axis and strain normal direction of the wave results in a reduction of the efficiency at long wavelengths according to the cosine function of the enclosed angle. However, the stretching of the apparent wavelength along the fiber axis, which is proportional to the scans of the same angle, slows down the decrease of the transduction efficiency at short wavelengths. Thus, in this region, more inclination between fiber and wavefront may enhance the strain conversion, as the characteristics for N=2 and the 45° and 60° inclination of Figure 13 reveal.

#### 4.2.2. Wavelength Dependence of Circular Fiber Transducers

**Figure 14:**
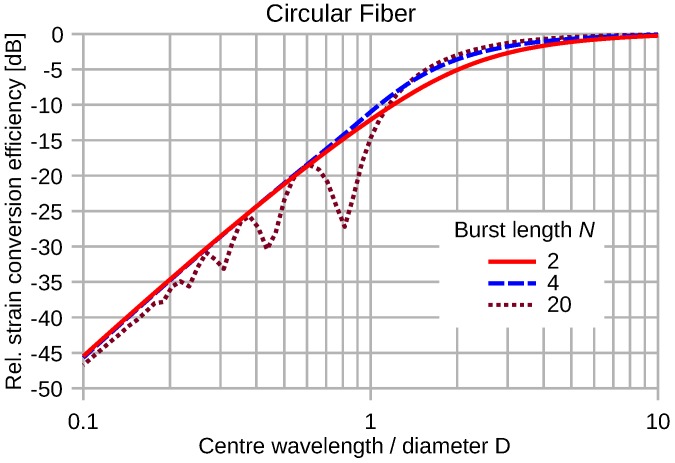
Normalized energy conversion of various sine-burst pulses by a circular fiber segment as a function of $\lambda_{M}/D$. The wave strain magnitude is kept constant.

**Figure 15:**
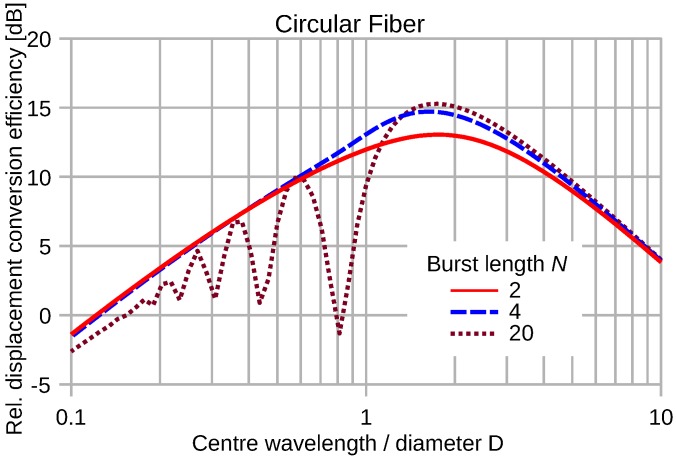
Normalized energy conversion of various sine-burst pulses by a circular fiber segment as a function of $\lambda_{M}/D$. The wave displacement magnitude is kept constant.

The response of a circular fiber transducer to elastic waves is omnidirectional. Figure 14 shows the normalized efficiency of the strain pulse conversion of a semicircular optical waveguide as a function of the center wavelength of an elastic plane wave pulse. Figure 15 depicts the efficiency of pulse energy transduction by circular fiber segments for constant wave strain magnitude.

Compared to the straight fiber transducer, the circular configuration shows a significantly worse transduction efficiency at short wavelengths. The notches of the circular characteristics do not coincide with the 1/N fractions of the center wavelength to diameter ratio.

The normalization of the conversion efficiency graphs hides the absolute values. For uniform strain or very long wavelengths, the output signal of a straight fiber transducer of length *D*, oriented in parallel to the propagation direction, exceeds that of a semicircular fiber of diameter *D* by a factor of 4/π. Because of the weighting function Equations (9) and (12), the output of the straight fiber is exceeded by a factor of π/2 by complementing the semicircle to a full turn.

## 5. Conclusions

The modeling of wave strain conversion using fiber-optic interferometry and compact strain pulses was successfully performed for several shapes of attached fiber segments at a broad range of ultrasonic wavelengths. The fiber-intrinsic conversion of wave strain demands for a finite length of rigid mechanical coupling between the optical fiber and the investigated body. This interaction length determines, on the one hand, the magnitude of the conversion efficiency and, on the other hand, its frequency dependence. In the time domain, distorted transduction takes place whenever the interaction length approaches the order of magnitude of the wavelength of the elastic perturbation. Small-scale FEM models are well suited to characterize distortions owing to distributed transduction. The shown examples of signal distortions were obtained with FEM analysis. The frequency dependence of pulse energy conversion, as well as the spatial conversion characteristics were derived from analytical models of the fiber-based strain transduction. The results are typical for all detection techniques of wave strain if the involved transducer employs a distributed conversion that extends over a significant fraction of a half wavelength. Further extensions of the presented analytical approach may comprise the transduction of dispersive elastic waves. Moreover, the interaction of circular crested elastic waves with circular, as well as arbitrarily-oriented straight fiber segments will be of substantial interest.
